# Effectiveness of a hospital intelligent financial system based on AI and RPA

**DOI:** 10.3389/fpubh.2026.1945091

**Published:** 2026-10-15

**Authors:** Jindong Zhao, Jie Zhang, Licai Pan

**Affiliations:** Department of Financial Management, Shandong Cancer Hospital and Institute, Shandong First Medical University and Shandong Academy of Medical Science, Jinan, Shandong, China

**Keywords:** application, artificial intelligence, financial systems, hospital, robotic process automation

## Abstract

**Background:**

Hospital financial management is facing ongoing challenges. Although artificial intelligence (AI) and robotic process automation (RPA) have shown their potential, there is still a lack of empirical evidence of the effectiveness of the integration of the two in the financial operations of hospitals. This study aims to evaluate the application effect of a set of an AI-RPA integrated intelligent financial system in a large hospital in China.

**Methods:**

This single-center, non-randomized, uncontrolled before-and-after study was carried out in a large tertiary A general hospital in China from January to December 2025. As an intervention, the intelligent AI-RPA financial system launched in June 2025 consists of four levels: data governance, business processing, technology application and risk control. The study compared the 6 months before the system was launched and the 6 months after the system was launched, in terms of operational efficiency (daily data collection time, report generation time, and automated processing time per transaction), data quality and risk control (data docking success rate, data entry error rate, logical conflict correction rate, and risk prediction accuracy for medical insurance violations) and health-care staff satisfaction.

**Results:**

After the system went online, the daily data collection time was shortened from 132.5 to 18.6 min (*P* < 0.001); the report generation time was shortened from 58.4 to 5.8 min (*P* < 0.001); the automated processing time of each transaction was reduced from 3.5 to 1.9 s (*P* < 0.001). Data interfacing success rate increased from 94.2 to 99.7%, data entry error rate dropped from 3.5 to 0.6%, logic conflict correction rate rose from 78.1 to 98.5%, and risk prediction accuracy for insurance violations improved from 72.1 to 89.3%. Total staff satisfaction score improved from 30.1 to 43.1 (*P* < 0.001).

**Conclusion:**

This study provides preliminary empirical evidence that the AI-RPA integrated intelligent financial system was associated with improvements in operational efficiency, data quality, risk control, and user satisfaction in a large Chinese hospital.

## 1 Introduction

As the global digital transformation of health-care continues to deepen, the concept of the smart hospital has expanded from early clinical informatics applications to a comprehensive smart service system covering the entire spectrum of operational management (1). As the core hub of hospital operation and management, the intelligent

upgrading of financial management systems directly determines the efficiency of medical resource allocation and profoundly influences the public welfare orientation and sustainable development capacity of the health-care system (2). Internationally, developed countries such as the United States and Italy have launched intelligent financial reforms with artificial intelligence (AI), robotic process automation (RPA) and blockchain as the core to meet the challenges of medical insurance payment pressure, cost increases and compliance requirements caused by population aging (3, 4). In contrast, although Chinese hospitals have made significant progress in the modernization of financial management, they still generally face structural problems such as data isolation, process fragmentation, heavy manual workload and risk detection delay (5). Against the backdrop of the new health-care reform, with the full implementation of policies such as diagnosis-related groups (DRG)/diagnosis-intervention packet (DIP) payment, zero mark-up on drugs and consumables, and tiered diagnosis and treatment, the revenue structure of public hospitals has fundamentally changed, with pressure on both income and expenditure. In terms of cost accounting, budget execution and performance evaluation, the need for refined management has become unprecedentedly urgent. Traditional hospital information system (HIS) or enterprise resource planning (ERP) systems lack dynamic adaptability and intelligent decision-making capabilities, and it is difficult to cope with high-frequency, multi-dimensional, and strictly regulated financial operating environments (6).

The integration of AI and RPA has opened up a breakthrough technical path for the restructuring of the hospital's financial model. RPA has the characteristics of non-invasive deployment, high process simulation fidelity and round-the-clock operation, which can efficiently replace highly repetitive and rule-based manual operations such as reimbursement audit, invoice verification, voucher generation, and bank-enterprise reconciliation (7). AI gives the system cognitive ability to perceive unstructured information, understand business semantics, predict trends and detect abnormalities (8). The deep integration of AI and RPA is not a simple superposition; on the contrary, AI empowers RPA and builds an intelligent financial system with self-learning and self-optimization capabilities.

However, the existing literature mainly focus on the application of AI in mainstream fields such as medical imaging diagnosis (9), clinical decision-making support (10), and public health forecasting (11), as well as the preliminary exploration of using RPA to optimize medical operational processes (12). There is still a lack of evaluation research on the application effect of AI and RPA combined technology in medical financial operation management. Most studies are limited to reports on the functional verification of a single technology module or the development of individual systems, and lack systematic empirical tests based on real hospital operation data and multi-dimensional performance indicators (7, 12). More importantly, hospital financial management has significant industry-specific characteristics: diverse sources of funds, strict budget constraints, strict regulatory framework, and the need to maintain a dynamic balance between public welfare goals and operational efficiency (13). These characteristics make it difficult to directly transplant general-purpose financial robots, specifically manifested in: insufficient robustness in identifying heterogeneous medical invoices, weak adaptability to clinical-financial composite processes, and lack of adaptability to dynamic compliance rules in the case of multi-policy parallel. Therefore, how to design an AI-RPA integration architecture tailored to the actual pain points of Chinese hospitals and scientifically evaluate its input and output, process performance, risk control and human-computer collaboration effects in real scenarios has become a core research topic in the intersection of medical management informatization and health policy.

To address these gaps, this study designs and implements a four-layer AI-RPA financial system covering the full hospital finance process and tailored to Chinese hospital finance. Using a single-center, non-randomized, uncontrolled before-and-after design, we evaluate it in a large tertiary A hospital in China over 6 months before and after implementation. Combining system logs, alert records, and a Technology Acceptance Model (TAM)-based staff survey, we assess multi-dimensional outcomes and examine whether the system was associated with changes in a complex medical operation environment. To our knowledge, this is among the first systematic evaluations of an integrated AI-RPA system in a large Chinese hospital, offering empirical evidence beyond technical feasibility and a transferable architecture and evaluation framework for hospital financial transformation.

## 2 Methods

### 2.1 Study design

This study adopts a single-center, non-randomized, uncontrolled before-and-after design to evaluate the application effect of a hospital intelligent financial system integrating AI and RPA technologies. The research was carried out in a large tertiary A general hospital in China from January 2025 to December 2025. The intelligent financial system was officially launched in June 2025 as the intervention point. The study compared the 6-month pre-intervention period with the 6-month post-intervention period. No other systems, staffing, or policy changes occurred between the two periods.

The study protocol was reviewed and approved by the Institutional Ethics Committee of the Cancer Hospital of Shandong First Medical University (No. SDTHEC2022010025). This study involved the evaluation of a hospital intelligent financial system using de-identified operational data and system logs, and did not involve the use of any clinical isolates, human biological samples, or patient-derived materials. The studies were conducted in accordance with the local legislation and institutional requirements.

### 2.2 Components of hospital intelligent financial systems based on the AI and RPA

The system consists of data governance, business processing, technology application, and risk control (Figure 1).

**Figure 1:**
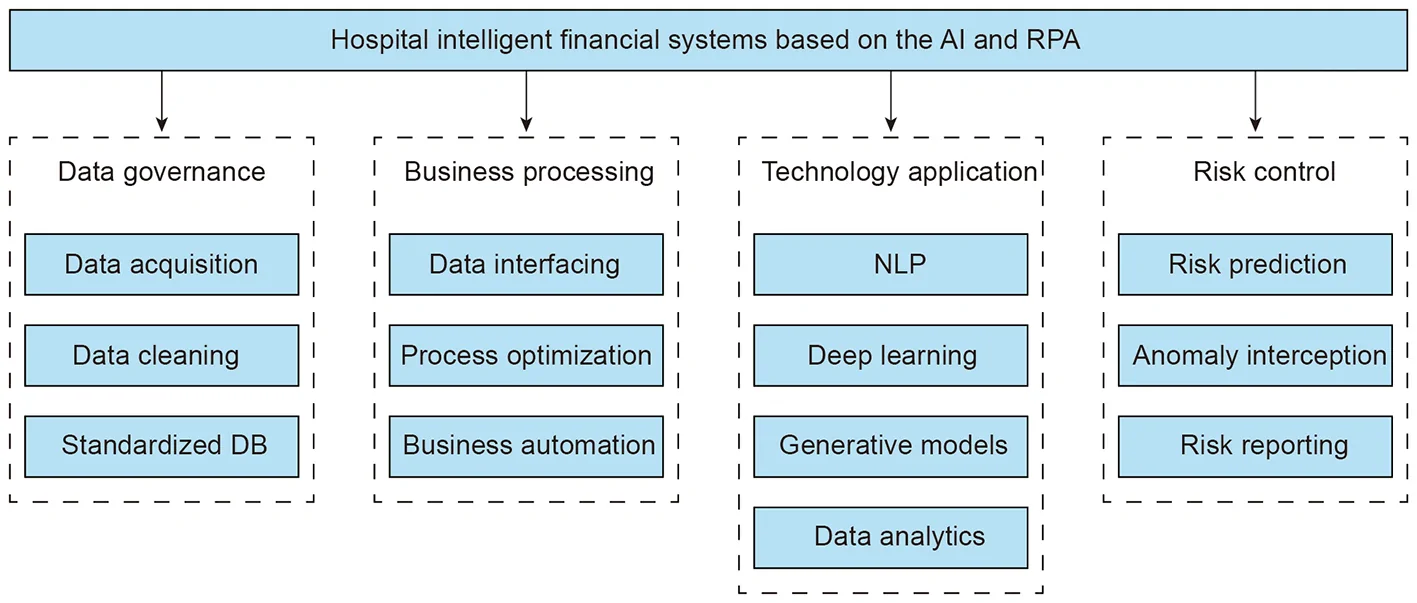
Components of hospital intelligent financial systems based on the AI and RPA.

(1) Data governance: this layer collects, cleans, and integrates data output from the business processing layer, then stores them in a standardized database. The data acquisition module automatically retrieves financial data from heterogeneous platforms such as ERP and banking systems and performs preliminary sorting based on preset robotic rules. The data cleaning module, leveraging AI-RPA analytical capabilities, identifies and handles missing values, duplicates, and logical conflicts. Through intelligent filling, deduplication, and error correction, it performs data validation and format conversion for structured, semi-structured, and unstructured data.
(2) Business processing: this layer focuses on financial data interfacing, process optimization, and business automation. In data interfacing, it establishes transmission channels and incorporates automated customer service tools. Upon receiving user business requests, the system retrieves relevant data from the data governance layer and provides immediate feedback via RPA robots. In process optimization, the AI-RPA components identify bottlenecks and redundant links in business processes, automatically reorganizing and reengineering them. In business automation, the system simulates manual operations to execute highly repetitive, rule-based financial tasks, including data entry, document approval, and report generation. Simultaneously, by learning and reasoning from historical business logic of similar tasks, the system derives and executes automated processing solutions for analogous businesses.
(3) Technology application: this layer integrates natural language processing (NLP), deep learning, generative models, and data analytics. The NLP module performs text cleaning, tokenization, and semantic vector techniques to understand the deep semantics of financial texts and complete human-computer language conversion. The deep learning module, relying on representation learning of deep neural networks, performs unsupervised, or semi-supervised learning on massive financial data, including hospital revenue, expenditure, and budget execution, to uncover latent patterns and execute complex financial tasks. Generative models produce high-fidelity simulated financial data, reports, and decision-making scenarios based on historical financial data and business rules. Data analytics techniques, from multidimensional perspectives such as revenue sources, cost structure, and asset operations and combined with external market trends, use AI-RPA to predict future hospital revenue, costs, and profits.
(4) Risk control: this layer covers risk prediction, anomaly interception, and risk reporting. In risk prediction, the system integrates multi-source data, including medical insurance reimbursement, financial revenue and expenditure, and contract performance, to construct risk prediction models. It identifies potential financial risks, such as insurance violations and false reimbursements, in advance and alerts managers. In anomaly interception, the system continuously learns from risk data collected by RPA, dynamically optimizes recognition rules, and intercepts abnormal financial operations in real time. In risk reporting, the system implements full-process, full-dimension control over financial risks and automatically generates risk reports.

### 2.3 Operational workflow of hospital intelligent financial systems based on the AI and RPA

The core workflow of the hospital intelligent financial system is illustrated in Figure 2. An RPA automated script simulates manual operation, including logging in to the system, browsing the menu, selecting the date range and exporting files, so as to regularly extract financial data from multiple source systems (including ERP systems, banking systems, pharmacy systems, human resources platforms, and research management systems). These scripts perform scheduled extraction tasks at predefined intervals (hourly or daily) to collect transaction records, account balances, purchase orders, payroll data, and research funding information. Subsequently, the obtained raw data will be entered into the data cleaning module. In this module, the missing value interpolation is first carried out, that is, filling empty fields through rule-based policies (including default values or adjacent records). Next, duplicate records are cleared, and the same entries are identified and deleted according to unique identifiers (such as transaction ID or combination field matching). Then carry out logical error correction to solve the inconsistency problem, especially when the expected value should be positive but there is a negative value, or the date mismatch between the invoice and the payment record. Finally, all data is converted into a unified format through standardized data conversion: date standardization to YYYY-MM-DD format, monetary unit standardization, and coding conversion (such as mapping department names to standard cost center codes). After these cleaning steps, the processed data will be stored in the central database as cleaned data.

**Figure 2:**
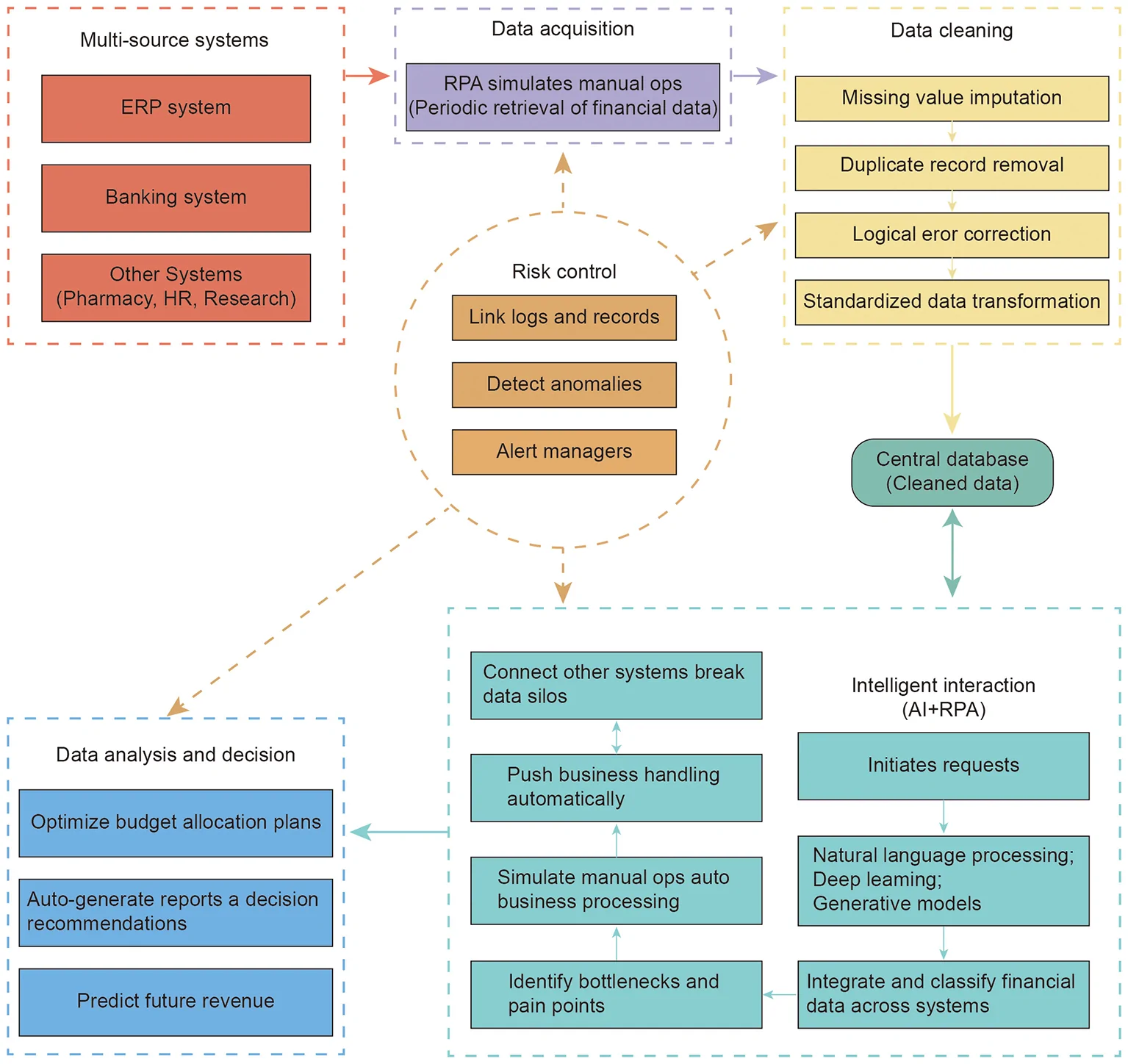
Operational workflow of hospital intelligent financial systems based on the AI and RPA.

In the intelligent interaction stage, the financial manager or authorized person initiates business requests through the system interface. The system uses NLP technology to parse unstructured text in request descriptions or attachments, uses deep learning to identify patterns in historical financial transactions, and generates draft output with the help of generative models, including budget summaries or difference descriptions. These technologies enable the system to integrate and classify financial data from different systems, thus connecting other systems and breaking down data islands. The system will automatically push the business processing tasks to the relevant users, such as transferring the reimbursement application to the corresponding approver, or sending a budget warning to the department head. The system performs automated business processing by simulating manual operations: matching invoices with purchase orders, calculating the remaining budget balance, and marking transactions that deviate from the historical normal. In the process, the system will identify bottlenecks and pain points, such as frequent manual re-entry steps, long waiting times between approvals, or repeated data inconsistencies from specific source systems. Through data analysis and decision-making functions, the system redistributes funds with low utilization to overspending departments, so as to optimize the budget allocation plan; automatically generate reports (such as monthly financial summaries or DRG cost statements); make decision-making suggestions (such as proposed cost control measures); and use time series prediction models to predict Future income.

At the same time, the risk control mechanism operates in real time in all early stages. The system can detect three types of anomalies: missing values with incomplete transaction records, duplicates (such as the same invoices submitted multiple times), and logical errors (including reimbursement amount exceeding the contract limit or account code mismatch, etc.). Once an abnormality is detected, the system will associate the operation log (user ID, access time, and modified data field) with data processing records (source file name, applied data cleaning steps, and output timestamp) to locate the root cause. This association will generate an audit track to determine whether the error is caused by a configuration error in the source system, an RPA script failure, or an input error. The system records each detection event and the root cause it traces back to in a dedicated log, so as to complete the closed-loop workflow from data collection to risk resolution.

### 2.4 Evaluation indicators

The evaluation indicators were defined and analyzed as follows. All objective indicators were automatically collected from complete system logs and alert records during the 6 months before and after system launch, forming a pre-post comparison. Daily data acquisition time was defined as the total time in minutes taken by the system to automatically retrieve transaction records, account balances, and other data from ERP, banking, and peripheral systems. It was calculated as the sum of all data retrieval durations within one calendar day. The unit of analysis was one calendar day. Report generation time was the average time in minutes taken by the system to automatically generate financial reports and budget execution analysis reports (14). It was calculated as the total generation time divided by the number of reports generated. The unit of analysis was one generated financial or budget execution report. Automated processing time per transaction was the average response time in seconds from receiving a user business request to completing automated processing and returning results. It was calculated as the total processing time divided by the number of transactions. The unit of analysis was one user business transaction. Data interfacing success rate was the percentage of successful cross-system data call requests out of total call attempts. It was calculated as (number of successful cross-system data call requests/total number of cross-system data call attempts) × 100%. The unit of analysis was one cross-system data call attempt. Data entry error rate was the percentage of erroneous data entries remaining after automatic acquisition and cleaning relative to total data entries. It was calculated as (number of erroneous data entries after automatic acquisition and cleaning/total number of data entries) × 100%. The unit of analysis was one data entry. Logic conflict correction rate was the percentage of automatically corrected logic conflicts out of total detected logic conflicts (15). It was calculated as (number of automatically corrected logic conflicts/total number of detected logic conflicts) × 100%. The unit of analysis was one detected logic conflict. Risk prediction accuracy for insurance violations was the percentage of correctly identified medical insurance violation risk events out of all actual violation events (16). It was calculated as (number of correctly identified medical insurance violation risk events/total number of actual violation events) × 100%. The unit of analysis was one actual insurance violation event.

Health-care staff satisfaction was assessed with a TAM-based scale encompassing perceived usefulness, perceived ease of use, and attitude toward use. Items were adapted from a validated TAM instrument and reworded to fit the intelligent financial system context (e.g., replacing “electronic medical record system” with “intelligent financial system”) (17). All items were rated on a 5-point Likert scale (1 = strongly disagree to 5 = strongly agree). Anonymous questionnaire surveys were administered to financial managers and health-care staff in relevant clinical departments before and after the intervention. To minimize response bias, participation was voluntary, standardized instructions were provided, and no personal identifiers or performance-related information were collected. Only participants who completed the questionnaire both before and after the intervention were included in the analysis.

### 2.5 Statistical analysis

All statistical analyses were performed using SPSS 27.0. Continuous variables conforming to normal distribution were expressed as mean ± standard deviation (x ± s). Given the single-center, non-randomized, uncontrolled before-and-after design, we used pre-post comparative analyses. Levene's test was used to confirm homogeneity of variances. Independent-samples *t*-tests were used for operational efficiency, data-quality, and risk-control indicators. Paired *t*-tests were used for TAM satisfaction scores. Categorical data were presented as *n* (%) and compared using the chi-square test. In all analyses, a two-tailed *P* < 0.05 was considered statistically significant.

## 3 Results

### 3.1 System operational efficiency

The analysis included 181 daily observations, 1,258 generated reports, and 2,715,310 processed transactions in the pre-intervention period, and 184 daily observations, 1,315 generated reports, and 2,802,120 processed transactions in the post-intervention period. Monthly aggregated trends are presented in Supplementary Table 1. After the implementation of the intelligent financial system, all operational efficiency indicators showed statistically significant improvements compared to the pre-intervention period (Table 1). The daily data acquisition time decreased from 132.5 ± 18.3 to 18.6 ± 3.1 min (*P* < 0.001), representing an 85.9% reduction. Report generation time was shortened from 58.4 ± 5.7 to 5.8 ± 0.9 min (*P* < 0.001), a reduction of 90.1%. The automated processing time per transaction decreased from 3.5 ± 0.2 to 1.9 ± 0.1 s (*P* < 0.001), a reduction of 45.7%.

**Table 1:** Comparison of system operational efficiency indicators between pre-intervention and post-intervention.

Indicator	Pre-intervention (Jan-Jun 2025)	Post-intervention (Jul-Dec 2025)	*t*	*P*
Daily data acquisition time (min)	132.5 ± 18.3	18.6 ± 3.1	83.224	< 0.001
Report generation time (min)	58.4 ± 5.7	5.8 ± 0.9	330.363	< 0.001
Automated processing time per transaction (s)	3.5 ± 0.2	1.9 ± 0.1	11,939.724	< 0.001

### 3.2 Data quality and risk control

The pre-intervention period included 1,630,218 cross-system data call attempts, 764,256 data entries, 42,937 detected logic conflicts, and 218 actual insurance violation events. The post-intervention period included 1,706,258, 813,467, 45,210, and 206 observations, respectively. Monthly aggregated trends are presented in Supplementary Table 1. The intelligent financial system also demonstrated marked improvements in data quality and risk control capabilities (Table 2 and Figure 3). The data interfacing success rate increased from 94.2% pre-intervention to 99.7% post-intervention, a gain of 5.5 percentage points. The data entry error rate declined from 3.5 to 0.6%, a reduction of 2.9 percentage points. The logic conflict correction rate rose from 78.1 to 98.5%, an improvement of 20.4 percentage points. Most notably, the risk prediction accuracy for insurance violations increased from 72.1 to 89.3%, representing a 17.2 percentage point enhancement.

**Table 2:** Comparison of data quality and risk control indicators between pre-intervention and post-intervention.

Indicator	Pre-intervention (Jan-Jun 2025)	Post-intervention (Jul-Dec 2025)	Change
Data interfacing success rate (%)	94.2	99.7	+5.5
Data entry error rate (%)	3.5	0.6	−2.9
Logic conflict correction rate (%)	78.1	98.5	+20.4
Risk prediction accuracy for insurance violations (%)	72.1	89.3	+17.2

**Figure 3:**
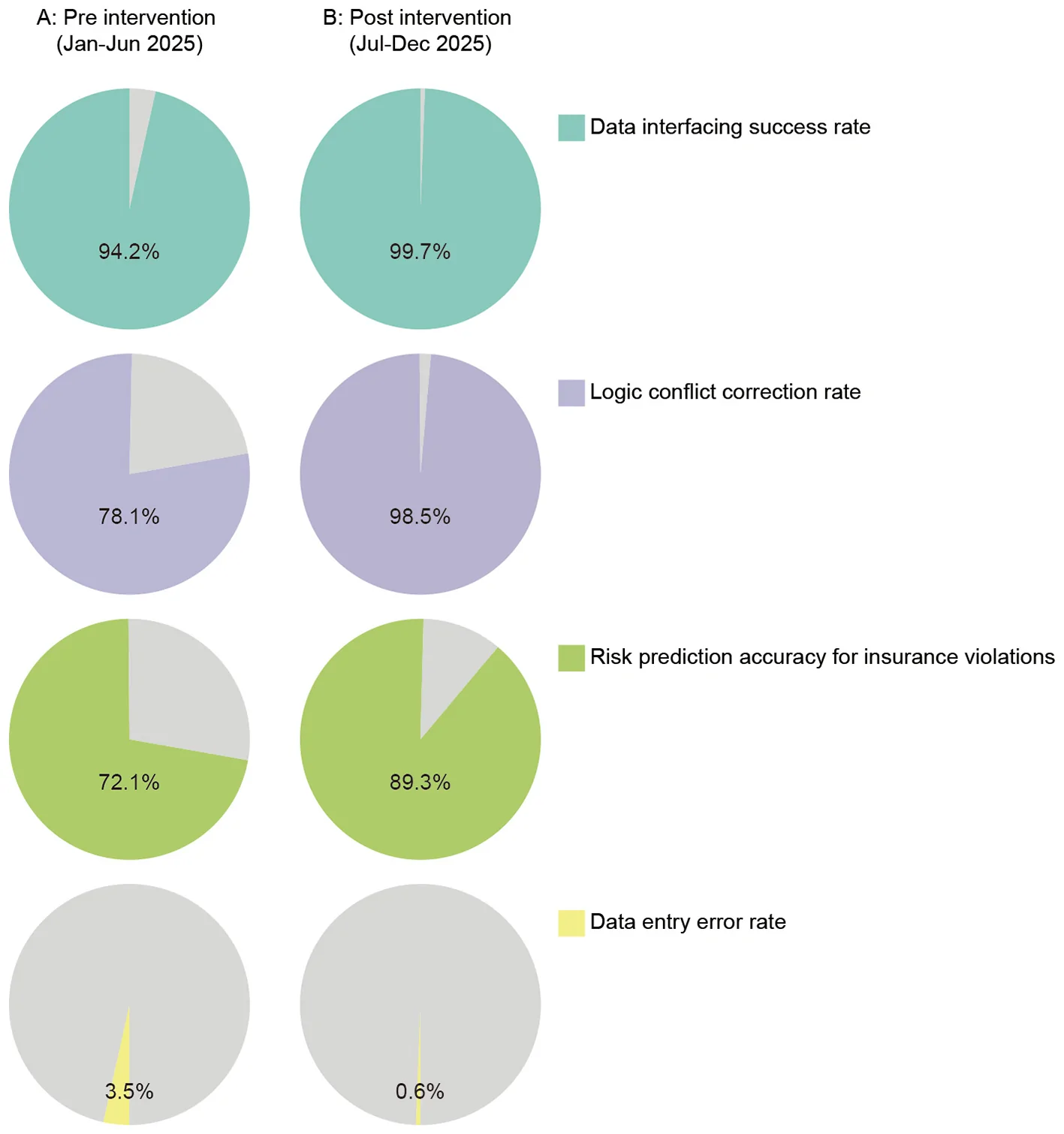
Improvement magnitudes across data quality and risk control indicators.

### 3.3 Health-care staff satisfaction

A total of 104 health-care staff members completed the satisfaction survey before and after the intervention. As shown in Table 3, all dimensions of the TAM scale showed statistically significant improvements. The perceived usefulness score increased from 11.4 ± 2.9 to 16.1 ± 3.0 (*P* < 0.001). The perceived ease of use score rose from 10.5 ± 2.6 to 15.3 ± 2.6 (*P* < 0.001). The attitude toward use score improved from 8.2 ± 2.3 to 11.8 ± 2.3 (*P* < 0.001). The total satisfaction score increased from 30.1 ± 4.2 to 43.1 ± 4.5 (*P* < 0.001).

**Table 3:** Comparison of health-care staff satisfaction between pre-intervention and post-intervention (*n* = 104).

Indicator	Pre-intervention (Jan-Jun 2025)	Post-intervention (Jul-Dec 2025)	*t*	*P*
Perceived usefulness	11.4 ± 2.9	16.1 ± 3.0	−11.273	< 0.001
Perceived ease of use	10.5 ± 2.6	15.3 ± 2.6	−14.024	< 0.001
Attitude toward use	8.2 ± 2.3	11.8 ± 2.3	−10.937	< 0.001
Total score	30.1 ± 4.2	43.1 ± 4.5	−20.968	< 0.001

## 4 Discussion

In this single-center, non-randomized, uncontrolled before-and-after study, we designed and implemented a hospital intelligent financial system integrating AI and RPA, and evaluated its potential association with application effectiveness in a large tertiary A general hospital. After system implementation, daily data acquisition time decreased by 85.9%, report generation time by 90.1%, and automated processing time per transaction by 45.7%. Data interfacing success rate increased by 5.5 percentage points, data entry error rate dropped by 2.9 percentage points, logic conflict correction rate improved by 20.4 percentage points, and risk prediction accuracy for insurance violations rose by 17.2 percentage points. Health-care staff satisfaction across perceived usefulness, ease of use, and attitude toward use also showed substantial gains. These findings provide empirical evidence for the feasibility and effectiveness of the integration model of AI-RPA in complex hospital financial operations.

From the perspective of design, the proposed four-layer architecture-namely data governance, business processing, technology application, and risk control-may help address core structural problems in the financial management of Chinese hospitals, including data isolation, process fragmentation and risk detection lag. Compared with traditional HIS or ERP systems, which often have limited dynamic adaptability in this context, the integration of AI and RPA may provide non-invasive deployment and high-fidelity process simulation while enhancing cognitive ability (4). In the data governance layer, the intelligent supplementation of missing values, duplicate data removal and error correction of heterogeneous data sources may help mitigate the long-standing problem of the diversity of medical invoice formats. This is achieved through rule-based and uninterrupted RPA operations, potentially reducing common inconsistencies and delays in manual data integration (18). The business processing layer can automate rule-based tasks such as reimbursement audit and voucher generation, potentially reducing labor workload. Another potential advantage is reduced context switching, because RPA robots can connect to different platforms (19). The technical application layer integrates NLP and deep learning technology to analyse the semantics of financial texts and identify patterns in income and expenditure data. At the same time, the generative model supports scenario simulation. Importantly, deep learning modules can learn latent patterns from historical financial data, which may allow the system to handle novel but similar tasks without explicit reprogramming. This ability is generally lacking in traditional rule-based systems (20). The risk control layer provides real-time interception and predictive modeling for insurance violations, aligning with the strict regulatory requirements associated with the DRG/DIP payment reform. The improvement in risk prediction accuracy may be closely associated with the system's continuous learning mechanism, which can dynamically update detection rules based on newly captured violation patterns (21). Previous studies have shown that such adaptive mechanisms are crucial to maintaining effectiveness in a changing regulatory environment (20). Overall, this design has the potential to transform the financial system from a passive recording tool to an intelligent and self-optimized decision support platform.

From the perspective of practical application, the observed changes were substantial in this single-center setting. The daily data collection time has been shortened from more than 2 h to less than 20 min, suggesting that manual repetition may have been replaced by RPA scripts running around the clock. One possible explanation is that these scripts are executed in parallel on multiple data sources, thus avoiding man-made delays (19). In addition, the automatic cleaning layer further shortens the processing time after collection (22). In view of the increase in the frequency of report generation and the increasing requirements for detail under the “zero price increase” and “graded diagnosis” policies, it is particularly noteworthy that the report generation time has been shortened by 90.1%. This effect comes not only from automation, but also from the system's ability to pre-aggregate and incremental verification of data, thus turning report generation into a retrieval task instead of heavy calculations from the beginning (23, 24). The automated processing time of each transaction has been shortened from 3.5 to 1.9 s. Although the decrease is not large alone, it represents an increase of 45.7%; considering the high transaction volume of large hospitals, this increase will have a significant cumulative effect. This acceleration marks a fundamental shift from a sequential, human-paced workflow to a parallel, machine-optimized workflow, in which the integration of AI and RPA eliminates redundant interaction between subsystems (19). The success rate of data interface increased from 94.2 to 99.7%, indicating that the reliability of the system has been enhanced and the number of manual processing abnormalities has been reduced. The accuracy of insurance violation risk prediction improved from 72.1 to 89.3%, which may help support compliance with changing reimbursement rules. This improvement is mainly due to the ability of deep learning modules to capture non-linear interactions between multiple risk factors, such as surgery codes, drug costs, and days of hospitalization, which traditional rule-based audit methods inevitably ignore. Previous studies have shown that deep learning methods perform far better than rule-based methods in detecting complex multi-factor compliance violations (25). Finally, the significant improvement in “perceptual usefulness” and “ease of use” shows that this human-computer collaboration design that integrates automated customer service tools and instant feedback has successfully reduced the cognitive load of financial managers and clinical staff and reduced their resistance to technology adoption (26). The improvement of ease of use is particularly convincing, which shows that the interface and interaction logic of the system are highly compatible with the existing workflow, thus lowering the learning threshold and encouraging active adoption.

Several limitations should be acknowledged. First, the single-center study design limits the universality of the results of this study to other hospital environments with different organizational structures or regional policies. Secondly, the 6-month observation period is too short to assess the long-term stability of the system, the drift of the AI model and the continuous acceptance of users. Third, this study does not cover potential negative effects, such as the deterioration of the skills of financial personnel due to over-reliance on automation, and the cost of early investment and maintenance. Fourth, the satisfaction data were obtained through self-reported anonymous questionnaires, which may be susceptible to response bias, including social desirability bias and expectation bias. Fifth, uncontrolled before-and-after design cannot fully exclude secular trends, seasonal case-mix differences, staff learning effects, or other concurrent changes. Future studies should also evaluate implementation and maintenance costs, potential skill degradation, and training needs, and should use qualitative methods, such as interviews with financial managers, to better understand human-computer collaboration. After longer-term validation, more advanced AI approaches, such as reinforcement learning for dynamic budget optimization, could be explored.

## 5 Conclusion

In this single-center, non-randomized, uncontrolled before-and-after study, the AI-RPA integrated intelligent financial system was associated with improvements in operational efficiency, data quality, risk control, and user satisfaction in a large Chinese hospital. The four-layer structure may offer a technically feasible and locally adaptable approach to persistent challenges such as data islands, process fragmentation, and delayed compliance monitoring in hospital financial management. Further multi-center, long-term, and controlled studies are needed to confirm these findings and assess sustainability, cost-effectiveness, and potential unintended effects.

## Data Availability

The original contributions presented in the study are included in the article/Supplementary material, further inquiries can be directed to the corresponding author.
