# Supplementary material for: Effectiveness of a hospital intelligent financial system based on AI and RPA
Source: Front Public Health. 2026 Oct 15;14:1945091. doi: 10.3389/fpubh.2026.1945091 (PMC13717361; doi:10.3389/fpubh.2026.1945091)
Supplement: Supplementary file 1 [file Table_1.docx]

**Supplementary Table S1.** Monthly trends before and after implementation of the AI-RPA integrated intelligent financial system in January-December 2025.

| Month | Daily observations | Reports generated | Transactions processed | Cross-system data call attempts | Data entries | Detected logic conflicts | Actual insurance violation events |
| --- | --- | --- | --- | --- | --- | --- | --- |
| Jan 2025 | 31 | 198 | 442318 | 271436 | 117218 | 7042 | 32 |
| Feb 2025 | 28 | 223 | 451976 | 261902 | 132543 | 6868 | 41 |
| Mar 2025 | 31 | 207 | 453105 | 282315 | 129809 | 7453 | 36 |
| Apr 2025 | 30 | 222 | 462744 | 271648 | 130106 | 7261 | 40 |
| May 2025 | 31 | 199 | 451893 | 272064 | 127672 | 7149 | 32 |
| Jun 2025 | 30 | 209 | 453274 | 270853 | 126908 | 7164 | 37 |
| Jul 2025 | 31 | 211 | 467215 | 284512 | 135612 | 7528 | 30 |
| Aug 2025 | 31 | 229 | 466842 | 284106 | 135408 | 7541 | 39 |
| Sep 2025 | 30 | 206 | 457508 | 275013 | 128879 | 7436 | 31 |
| Oct 2025 | 31 | 232 | 466974 | 294367 | 139267 | 7932 | 38 |
| Nov 2025 | 30 | 219 | 477328 | 284887 | 137734 | 7239 | 34 |
| Dec 2025 | 31 | 218 | 466253 | 283373 | 136567 | 7534 | 34 |
